# ALKBH5-Mediated m^6^A Modification of A20 Regulates Microglia Polarization in Diabetic Retinopathy

**DOI:** 10.3389/fimmu.2022.813979

**Published:** 2022-03-01

**Authors:** Tingting Chen, Wenhui Zhu, Congyao Wang, Xia Dong, Fenfen Yu, Yihua Su, Jingwen Huang, Lijun Huo, Pengxia Wan

**Affiliations:** Department of Ophthalmology, The First Affiliated Hospital, Sun Yat-sen University, Guangzhou, China

**Keywords:** diabetic retinopathy, microglia polarization, A20, m^6^A modification, ALKBH5

## Abstract

**Background:**

To investigate the role of microglia polarization in the pathogenesis of diabetic retinopathy, and study the mechanism of ALKBH5-mediated m^6^A modification of A20 of retinal microglia polarization.

**Methods:**

Diabetics rats were constructed and the M1/M2 polarization of retinal microglia was determined using immunofluorescence, flow cytometry, and quantitative real-time PCR (qRT-PCR). Glucose at different concentrations was added to treat the microglia, and the polarization rate was detected. RNA sequencing was performed to identify the differentially expressed gene in glucose treated microglia, and A20 expression was confirmed by qRT-PCR and western blotting. Lentiviruses encoding shRNA for A20 or overexpressing A20 were constructed to clarify the role of A20 in microglia polarization *in vitro* and vivo. N^6^-methyladenosine (m^6^A) modification level and degradation rate of A20 were determined and m^6^A related proteins were detected.

**Results:**

Diabetics rats showed a higher M1 polarization rate but lower M2 polarization rate of retinal microglia. With the increase of glucose concentration, microglia tend to polarize into M1 inflammatory type rather than M2 anti-inflammatory type. Shown by RNA sequencing, glucose treated microglia showed a differentially expressed gene profile, which was enriched in kinds of inflammatory categories and pathways. A20 expression was lower in microglia with glucose treatment, which was demonstrated to negatively regulate the M1 polarization. Moreover, intraocular injection of A20-overexpression lentiviruses (OE-A20) rectified the enhanced M1 retinal microglia polarization of diabetes rats. The higher m^6^A modification level and faster degradation rate of A20 was observed in glucose treated microglia, which was mediated by m^6^A demethylase ALKBH5.

**Conclusion:**

Lower expression A20 resulted in the enhanced M1 polarization of retinal microglia in diabetic retinopathy, which was caused by ALKBH5 mediated m^6^A modification. This study may provide new perspectives on not only the pathogenesis but also the diagnosis and treatment for diabetic retinopathy.

## Introduction

The number of persons living with diabetes mellitus (DM) is projected to increase to 366 million in 2030 globally (1). Diabetic retinopathy (DR), one of the most common complications of diabetes mellitus, is the leading cause of blindness and visual impairment among working-age adults global (2, 3).

Previously, DR was considered a kind of microvascular abnormality and neurodegeneration, nevertheless, inflammation was recently perceived as central to pathogenic processes in DR with the development of researches (4, 5). Microglia cells are the tissue-resident macrophages in the retina, which contributed greatly to retinal tissue homeostasis (6, 7). Currently, the unbalanced polarization of microglia-induced retinal inflammation was assumed to be one of the key pathogenesis of DR (8, 9). However, the mechanism of microglia polarization under high glucose conditions still needs to be addressed.

A20, also named tumor necrosis factor-α induced protein 3 (TNFAIP3), is a ubiquitin-related enzyme possessing both deubiquitinating and ubiquitinating abilities (10). Known as an anti-inflammatory molecule, A20 shows inhibitory effects and prevents several signal pathways from activation inflammation, which including the Toll-like receptor signal pathway and nuclear factor κB (NF-κB) signal pathway (11, 12). Research has demonstrated that abnormal expression or dysfunction of A20 could lead to local and systemic inflammation *in vivo* (13, 14). Moreover, A20 was shown to contribute to the regulation of microglial function (15). The role of A20, nevertheless, is still unclear on the polarization of microglia in DR.

N^6^-methyladenosine (m^6^A) is the most abundant internal messager RNAs (mRNA) modification in mammalian cells (16). Previous studies have demonstrated m^6^A modification not only played an important role in regulating gene expression (17), but also been involved in the followed cell functions (18). Recent studies reported that m^6^A modification may contribute to the pathogenesis of diabetic retinopathy (19). The possibility exists in the mechanism of unbalanced M1/M2 polarization of microglia in DR.

In our study, we investigated the mechanism of unbalanced M1/M2 microglia polarization in DR and demonstrated that decreasing expression of A20, regulated by human AlkB homolog 5 (ALKBH5) mediated m6A modification, led to enhanced M1 inflammatory polarization of microglia under high glucose conditions in DR. This study may provide new insight into not only the pathogenesis but also the clinical intervention for DR.

## Material and Methods

### Study Approval

This study was approved by the Ethics Committee of the First Affiliated Hospital, Sun Yat-Sen University, Guangzhou, China. The experiments on mice were approved by the Institutional Animal Care and Use Committee of Sun Yat-Sen University, Guangzhou, China. All experimental procedures on mice were carried out in strict adherence to the rules and guidelines for the ethical use of animals in research.

### Animals

The male Sprague-Dawley rats (8 weeks old, 200-220 g) were purchased from the Laboratory Animal Center of Sun Yat-sen University. Streptozotocin (Sigma, USA) was given by intraperitoneal injection at a dose of 60 mg/Kg to induce diabetics rats, while the control rats were given by empty citrate buffer. One week after induction, those rats with blood glucose levels > 16.7 mmol/L for three times were considered as successful inducted diabetes. All the rats did not receive insulin during the experiments.

In the intraocular injection experiments, rats confirmed as the DM model (blood glucose levels > 16.7 mmol/L for three times) were anesthetized with an intraperitoneal injection of sodium pentobarbital (50 mg/Kg). A total of 10 ul DMEM with 1*10^9^ TU lentiviruses (A20-overexpression, OE-A20 group) or the same volume of DMEM with control lentiviruses (OE-NC group) was injected into the vitreous cavity using a 33-gauge needle. This treatment was performed one time per month, and the rats were sacrificed for further experiments at the 3 months.

### Tissue Harvesting and Retinal Microglia Isolation

At the 1, 2 and 3 months after streptozotocin injection, the diabetic rats were euthanized and their enucleated eyes were dissected to remove the cornea and lens. The retinal tissues obtained were successively fixed and embedded in paraffin for experiments. For retinal microglia isolation, the retina was gently separated from enucleated eyes using forceps and then was digested in collagenase A (1.5 mg/ml; Sigma, USA) with Deoxyribonuclease I (0.5 mg/ml; Thermofisher, USA) for 1 hour at 37°C. The single-cell suspension was generated by passing through 70 μm filters. The retinal microglia were isolated using CD11b/c MicroBeads (Miltenyi Biotec, Germany) according to the protocol for further experiments.

### Cell Culture and Treatment

Mouse microglia cell line BV2 was purchased from the BeNa Culture Collection (China). BV2 was cultured in Dulbecco modified Eagle medium (Gibco, USA) with 10% fetal bovine serum (FBS, Gibco, USA) at 37°C and with 5% CO_2_. The basal concentration of glucose in DMEM was 5.55 mM. When the culture reached 80-90% confluence, BV2 was seeded in 12-well plates at a density of 1.5×10^4^ cells/cm^2^. *In vitro* glucose treatment experiments, additional 10, 50 and 100 mM glucose were added in the medium to incubate with BV2 for 72 hours. Medium without extra glucose (0 mM) was treated as the control.

### Immunofluorescence Assay

Sections were deparaffinized, hydrated, and incubated in 1% Triton X-100/PBS. After antigen retrieval in citrate buffer and blocking in donkey serum, the sections were incubated with Anti-CD16 antibody (Abcam, UK) or Anti-CD206 antibody (Abcam, UK) at 4°C overnight. After washing by phosphate buffered saline (PBS) three times, the sections were incubated with fluorescein-conjugated secondary antibody (Abcam, UK) for 1 hour and then with DAPI for another 10 min. All images were obtained using an LSM 5 Exciter confocal imaging system (Carl Zeiss, Germany).

### Flow Cytometry

The isolated CD11b/c^+^ retinal microglia were incubated with anti-CD16 antibody (NOVUS, China) or anti-CD206 antibody (Invitrogen, USA) for 30 min at room temperature. After being washed with PBS for three times, the CD11b/c^+^ retinal microglia were respectively incubated with Mouse F(ab)2 IgG (H+L) PE-conjugated Antibody or Rabbit IgG PE-conjugated Antibody (R&D, USA) for 15 min at room temperature. Flow cytometry of BV2 was conducted using the Rat Anti-Mouse CD16/CD32 or Alexa Fluor^®^ 647 Rat Anti-Mouse CD206 (BD Pharmingen™, USA). All labeled cells were detected using BD Influx cell sorter (BD Biosciences, USA).

### Quantitative Real-Time PCR

Total RNA of the isolated CD11b/c^+^ retinal microglia from diabetes rats or BV2 were extracted using Trizol Reagent (Invitrogen, USA) according to the protocol. Reverse transcription assay was performed using PrimeScript™ reagent kits (TaKaRa, Japan) to obtain the cDNA. QRT-PCR, reactions were performed in LightCycler^®^480 PCR system (Roche, USA) using SYBR^®^ Premix Taq™ kits (TaKaRa, Japan). The relative expression levels were analyzed using the 2^-ΔΔCt^ method. Primers for each gene are shown in **Supplemental Table 1**.

### RNA Sequencing and Bioinformatics Analysis

RNA of BV2 treated with 50 mM glucose for 72 hours (GU group) and those without glucose (NC group) were extracted, and the cDNA libraries were constructed. RNA sequencing assays were performed using the BGISEQ-500 platform by the Beijing Genomics Institute. Data were filtered and the clean reads were mapped to a reference genome GRCh38/hg38. Differentially expressed genes with a fold change of >2.0 or <-2.0 and P values <0.05 were selected for further analyses. The bioinformatics analysis was performed using BGI Dr. Tom 2.0, including heatmap clustering, gene ontology (GO) analysis, Kyoto Encyclopedia of Genes and Genomes (KEGG) analysis, and protein-protein interaction (PPI) network.

### Western Blot

BV2 were lysed in RIPA lysis buffer (Thermo Fisher, USA) containing protease inhibitors. The lysates were centrifuged and the supernatant was obtained. Protein concentrations were determined using a Pierce BCA protein assay kit (Thermo Fisher, USA) and then were boiled with sample loading buffer (Thermo Fisher, USA). Equal amounts of protein were separated using sodium dodecyl sulfate-polyacrylamide gel electrophoresis and subsequently transferred to polyvinylidene fluoride (PVDF) membranes (Millipore, USA), which were then blocked in 5% skim milk and incubated with primary antibodies against GAPDH, A20, METTL3, METTL14, ALKBH5 or FTO (all from CST, USA) overnight. The PVDF membranes were incubated with horseradish peroxidase (HRP)-conjugated secondary antibody after washing. Immobilon Western Chemiluminescent HRP Substrate (Millipore, USA) was used to detect the specific antibody-antigen complexes. The mean intensity ratio was analyzed using ImagePro Plus 6.0.

### Lentivirus Construction and Transfection

The lentiviruses encoding a short hairpin RNA (shRNA, **Supplemental Table 1**) specific for A20 or ALKBH5, and the A20-overexpression lentiviruses and ALKBH5-overexpression lentiviruses were designed and synthesized by OBiO Technology Corp., Ltd (China). The inhibition and overexpression effects of the lentiviruses were confirmed by qRT-PCR and Western blot assays before experiments. Lentiviruses (10^9^ TU/mL) and 5 μg/mL polybrene were added into the medium and incubated with BV2 at a multiplicity of infection of 20. After 24 hours, medium containing lentiviruses were removed, and BV2 were cultured for another 3 days with 50 mM glucose. Functional experiments were performed as described subsequently. For *in vivo* experiment, OE-A20 and its control OE-NC lentiviruses for rats were also constructed.

### Enzyme-Linked Immunosorbent Assay (ELISA)

The culture supernatant of BV2 was collected, and the concentration of IL-1β, IL-6, TNF-α, IL-4, IL-10 and TGF-β were detected using the Quantikine ELISA kits according to the protocols (all from R&D, USA).

### m^6^A RNA Immunoprecipitation (RIP)

The m^6^A RIP assay was performed using the Magna MeRIP™ m6A Kit (Millipore, USA). Briefly, RNA was extracted and then chemically fragmented into fragments of no larger than 200 nucleotides. The RNA fragments were incubated with anti-m^6^A antibody- or IgG-conjugated Protein A/G magnetic beads at 4°C overnight. The magnetic beads were collected and the bound m^6^A-modified RNA was eluted for qRT-PCR analysis. Equal amounts of non-immunoprecipitated RNA fragments were used as the input control. The fold enrichment of each target gene was calculated as previously reported (20).

### RNA Stability Assays

Actinomycin D was added into the medium at a final concentration of 20 μg/ml, and BV2 were treated for 0, 1, 2, and 3 hours. RNA was then extracted and qRT-PCR was performed to detect the A20 expression. The turnover rate and half-life of A20 mRNA were calculated as described (21).

### Dual-Luciferase Reporter Assay

ALKBH5 expression vector and luciferase reporter vectors containing the C-terminal DNA fragment of ELMO1 or its mutant with mutations in m6A modification sites (A replaced by T) were synthesized. The possible m6A modification sites were predicted by the m6AVar database, and the top three mutant sites were chr6:137867497, chr6:137867811 and chr6:137876175. BV2 cells were seeded and then 1.5 μg luciferase reporter vector, 1.5 μg ALKBH5 vector, and 1.5 μg Renilla luciferase reporter vector were cotransfected into BV2 cells using the Lipofectamine 3000 Transfection Kit according to the manufacturer’s instructions. After 48 h of transfection, the luciferase activity was detected using the Dual-Luciferase^®^ Reporter Assay System Kit (Promega) according to the protocol. Relative Fluc/Rluc activity was calculated by normalizing the activity of firefly luciferase to that of Renilla luciferase.

### Statistical Analysis

All the results were determined based on at least three separate experiments containing at least triplicate samples. The two group comparisons were performed using a 2-tailed Student’s t-test, and comparisons of three or more different groups were performed by a one-way ANOVA, followed by Bonferroni’s *post hoc* comparisons. Data are expressed as the means ± standard deviations. Statistical analysis was performed with SPSS 16.0 (SPSS Inc.). P-values less than 0.05 were considered statistically significant.

## Results

### Increased M1 Polarization While Decreased M2 Polarization of Retinal Microglia in Diabetes Rats

**Figure 1:**
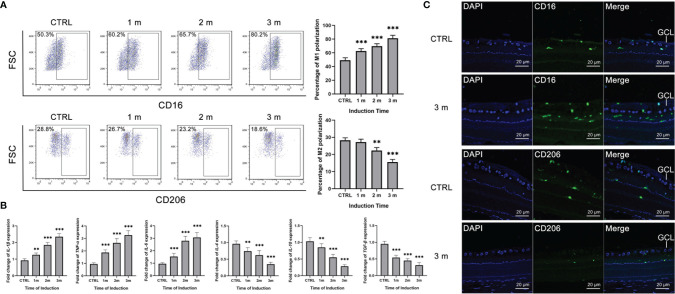
Increased M1 polarization but decreased M2 polarization of retinal microglia in diabetes rats. **(A)** The percentage of CD16^+^ M1 microglia increased from 0 to 3 months after STZ induction. The percentage of CD206^+^ M2 microglia decreased at the 2 and 3 months after STZ induction. **(B)** The mRNA levels of IL-1β, IL-6 and TNF-α increased from 0 to 3 months after STZ induction. The mRNA levels of IL-4, IL-10 and TGF-β decreased from 0 to 3 months after STZ induction. **(C)** The 3 months after STZ induction group have more CD16^+^ M1 microglia but fewer CD206^+^ M2 microglia compared to the control group. Values are presented as the mean ± SD. ** indicates P < 0.01, and *** indicates P < 0.001.

The diabetes rat models were constructed, and the microglia polarization in the retina was detected at 1, 2 and 3 months. Retinal microglia of the diabetes rats were isolated and the polarization rates were detected by flow cytometry. The M1 polarization percentage increased from 0 to 3 months, with the gradual decrement of the M2 polarization rate (**Figure 1A**). Besides, the M1 polarization markers, including IL-1β, IL-6 and TNF-α increased in the isolated retinal microglia from diabetes rats, with the decreasing M2 polarization markers such as IL-4, IL-10 and TGF-β (**Figure 1B**). Moreover, immunofluorescence results showed that the number of CD16^+^ M1 polarization retinal microglia increased at 3 months after STZ induction. Inversely, the number of CD206^+^ M2 polarization retinal microglia decreased compared to the control group (**Figure 1C**).

### Glucose Promoted M1 Polarization But Inhibited M2 Polarization of Microglia

**Figure 2:**
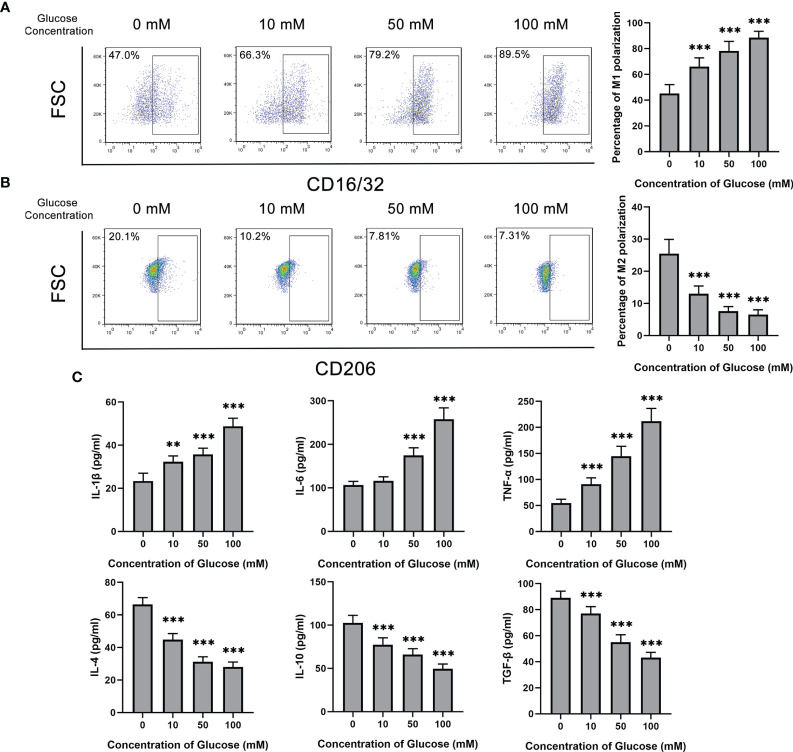
Glucose promoted M1 polarization but inhibited M2 polarization of microglia. **(A)** The percentage of CD16/32^+^ M1 microglia gradually increased with glucose treatment from the concentration of 0 to 100 mM. **(B)** The percentage of CD206^+^ M2 microglia gradually decreased with glucose treatment from the concentration of 0 to 100 mM. **(C)** The secretion levels of IL-1β, IL-6 and TNF-α up-regulated with the increase of glucose concentration, and the secretion levels of IL-4, IL-10 and TGF-β down-regulated with the decrement of glucose concentration. Values are presented as the mean ± SD. ** indicates P < 0.01, and *** indicates P < 0.001.

Glucose was added to treat BV2 cells at different concentrations from 0 to 100 mM. With the increase of glucose concentration, the M1 polarization rate of BV2 marked by CD16/32 showed a rising trend (**Figure 2A**). On the contrary, the percentage of M2 polarization decreased from about 20% without glucose stimulation to 7% in 100 mM glucose treatment (**Figure 2B**). Consistently, M1 or M2 microglia related cytokines show the same tendency. The concentration of IL-1β, IL-6 and TNF-α, which were originated from M1 polarization microglia, increased after glucose treatment. M2 polarization secreting cytokines, including IL-4, IL-10 and TGF-β, kept reducing with glucose treatment (**Figure 2C**).

### A20 Expression of Microglia Decreased With Glucose Stimulation

**Figure 3:**
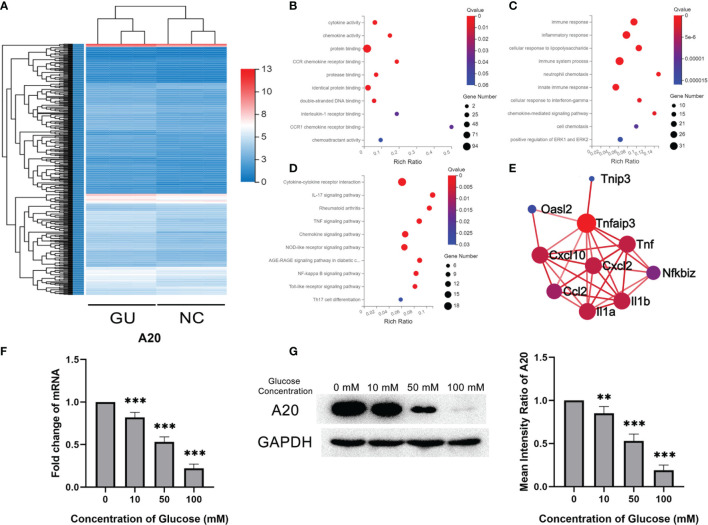
A20 expression of microglia decreased with glucose stimulation. **(A)** The heatmap of differentially expression gene profiles. **(B)** The top 10 enriched categories of molecular function in GO analysis. **(C)** The top 10 enriched categories of biological processes in GO analysis. **(D)** The top 10 enriched signal pathways of KEGG analysis. **(E)** The PPI network of differentially expressed mRNA. **(F)** The A20 expression at mRNA level decreased from 10 to 100 mM glucose treatment. **(G)** The A20 expression at protein levels decreased with 50 and 100 mM glucose treatment. Values are presented as the mean ± SD. ** indicates P < 0.01, and *** indicates P < 0.001.

The gene expression profile of BV2 treated with glucose was detected and compared with that of BV2 without glucose. A total of 484 differentially expressed genes were identified, with 90 up-regulated genes and 394 down-regulated genes (**Figure 3A**). Bioinformatics analysis was performed to investigate the deep information about the sequencing data. As determined by GO analysis, 484 differentially expressed genes were enriched in the cytokine activity, chemokine activity and protein binding in the molecular function category, and in the immune response, inflammatory response and immune system process in the biological process category (**Figures 3B, C**). KEGG pathway analysis showed that these differentially expressed genes were enriched in inflammatory-related signal pathways, such as cytokine-cytokine receptor interaction, IL-17 signaling pathway and TNF signaling pathway (**Figure 3D**). A20 (also named as TNFAIP3), enriched in the PPI network and related to kinds of inflammatory cytokines, was significantly lower in the glucose treatment than the control group (**Figure 3E**). Consistent with the RNA sequencing results, qPCR showed that A20 expression in BV2 gradually decreased with the increase of glucose concentration (**Figure 3F**). These results were also confirmed by the western blotting assays in protein levels (**Figure 3G**).

### A20 Negatively Regulated the M1 Polarization of Microglia

**Figure 4:**
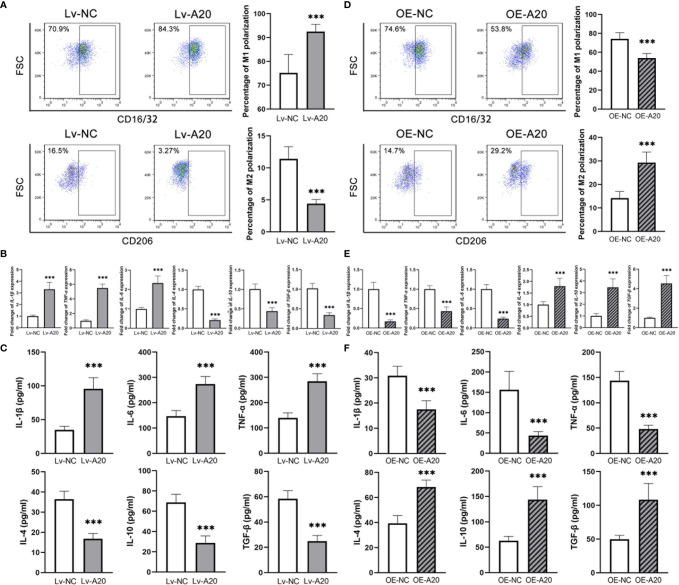
A20 negatively regulated the M1 polarization of BV2 microglia. **(A)** The percentage of CD16/32^+^ M1 microglia was higher in the Lv-A20 group than in the Lv-NC group. The percentage of CD206^+^ M2 microglia was lower in the Lv-A20 group than in the Lv-NC group. **(B)** The IL-1β, IL-6 and TNF-α expression at mRNA levels were higher in the Lv-A20 group than the Lv-NC group, and the IL-4, IL-10 and TGF-β expression at mRNA levels were lower in the Lv-A20 group than the Lv-NC group. **(C)** The IL-1β, IL-6 and TNF-α secretion levels were higher in the Lv-A20 group than the Lv-NC group, and the IL-4, IL-10 and TGF-β secretion levels were lower in the Lv-A20 group than the Lv-NC group. **(D)** The percentage of CD16/32^+^ M1 microglia was lower in the OE-A20 group than in the OE-NC group. The percentage of CD206^+^ M2 microglia was higher in the OE-A20 group than in the OE-NC group. **(E)** The IL-1β, IL-6 and TNF-α expression at mRNA levels were lower in the OE-A20 group than the OE-NC group, and the IL-4, IL-10 and TGF-β expression at mRNA levels were higher in the OE-A20 group than the OE-NC group. **(F)** The IL-1β, IL-6 and TNF-α secretion levels were lower in the OE-A20 group than the OE-NC group, and the IL-4, IL-10 and TGF-β secretion levels were higher in the OE-A20 group than the OE-NC group. Values are presented as the mean ± SD. *** indicates P < 0.001.

To clarify the effect of A20 in the microglia polarization, a lentivirus encoding a short hairpin RNA for A20 and an A20 overexpression lentivirus were constructed. The inhibitory and overexpression effect were confirmed in mRNA and protein levels (**Supplementary Figure 1**). After inhibiting A20 expression in BV2 cells treated with 50 mM glucose, the percentage of M1 polarization markedly increased compared to the Lv-NC group, with a significantly lower rate of M2 polarization (**Figure 4A**). Besides, the expression levels of M1 polarization markers were higher in the Lv-A20 group compared to the Lv-NC group, and those of M2 polarization markers in the Lv-A20 group were lower compared to the Lv-NC group (**Figure 4B**). The protein levels of IL-1β, IL-6 and TNF-α in the cultural supernatant of Lv-A20 transfected microglia were significantly increased. Moreover, Lv-A20 transfected microglia secreted lower levels of IL-4, IL-10 and TGF-β (**Figure 4C**). Microglia transfected with A20-overexpression lentiviruses showed stronger M2 polarization potential but weaker M1 polarization ability compared to the control group (**Figure 4D**). In terms of both the mRNA and the protein levels, the IL-1β, IL-6 and TNF-α expressions of the OE-A20 group were lower, and the IL-4, IL-10 and TGF-β expressions of the OE-A20 group were higher compared to the OE-NC group (**Figures 4E, F**).

### Intravitreal Injection of A20-Overexpression Lentiviruses Rectified Enhanced M1 Polarization of Retinal Microglia in Diabetes Rats

**Figure 5:**
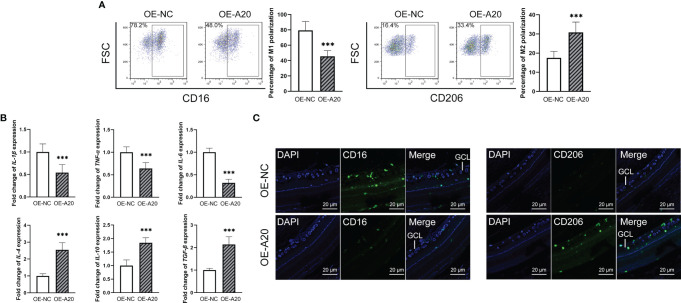
Intravitreal injection of A20-overexpression lentiviruses rectified enhanced M1 polarization of retinal microglia in diabetes rats. **(A)** The percentage of the CD16^+^ M1 microglia in the OE-A20 group was lower than that in the OE-NC group. The percentage of the CD206^+^ M1 microglia in the OE-A20 group was higher than that in the OE-NC group. **(B)** The mRNA levels of IL-1β, IL-6 and TNF-α were lower in the OE-A20 group compared to the OE-NC group. The mRNA levels of IL-4, IL-10 and TGF-β were higher in the OE-A20 group compared to the OE-NC group. **(C)** The OE-A20 group has less CD16^+^ M1 microglia but much more CD206^+^ M2 microglia compared to the OE-NC group. Values are presented as the mean ± SD. *** indicates P < 0.001.

To clarify the effect of A20 on the retinal microglia polarization of diabetes rats, OE-A20 or its control OE-NC was injected into the vitreous cavity. The ratio of CD16^+^ M1 retinal microglia in the OE-A20 group was lower than that in the OE-NC group, with the reverse tend of the CD206^+^ M2 retinal microglia (**Figure 5A**). The M1/M2 polarization marker genes showed consistent results (**Figure 5B**). Shown by immunofluorescence assay, diabetes rats receiving OE-A20 treatment have a smaller amount of CD16^+^ M1 polarization retinal microglia compared to the OE-NC treated group. Besides, the number of CD206^+^ M2 polarization retinal microglia was higher in the OE-A20 group than those in the OE-NC group (**Figure 5C**).

### Decreasing ALKBH5 Led to Lower A20 Expression Through m^6^A Modification in Microglia With Glucose Stimulation

**Figure 6:**
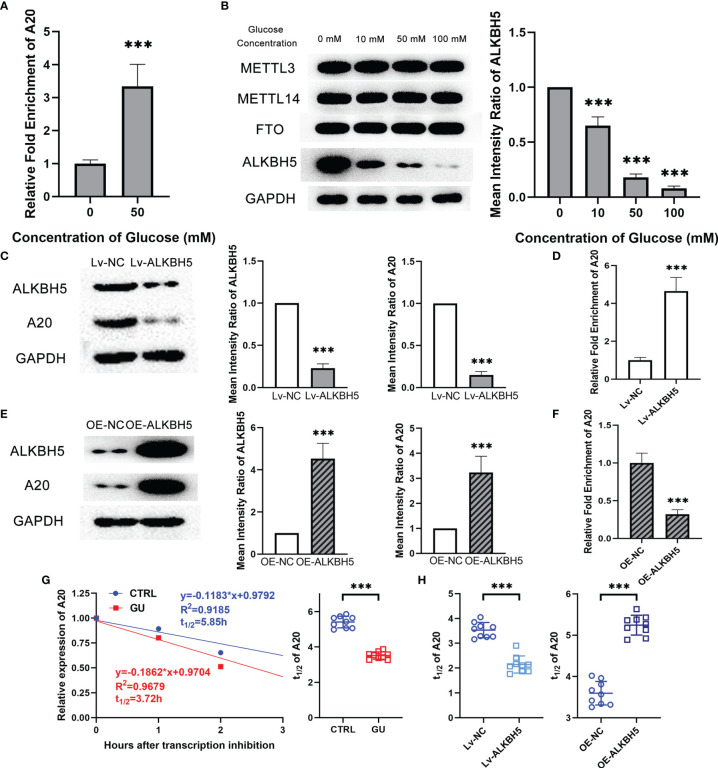
Decreasing ALKBH5 led to lower A20 expression through m^6^A modification in microglia with glucose stimulation. **(A)** The m^6^A modification level of A20 was higher in the glucose treatment group than in the control group. **(B)** ALKBH5 expression at protein level decreased with glucose treatment. The expression of METTL3, METTL14 and FTO kept unchanged. **(C)** Lv-ALKBH5 significantly inhibited the expression of both ALKBH5 and A20. **(D)** The m^6^A modification level of A20 was higher in the Lv-ALKBH5 group than in the Lv-NC group. **(E)** OE-ALKBH5 significantly promoted the expression of both ALKBH5 and A20. **(F)** The m^6^A modification level of A20 was lower in the OE-ALKBH5 group than in the OE-NC group. **(G)** The degradation rate of A20 mRNA shown by t_1/2_ value was higher in the glucose treatment group than in the control group. **(H)** Lv-ALKBH5 decreased the t_1/2_ value, and OE-ALKBH5 increased the t_1/2_ value of A20 mRNA. Values are presented as the mean ± SD. *** indicates P < 0.001.

Recently, researchers demonstrated that m^6^A modification affected the mRNA stability and its subsequent expression (22). To investigate the reason for the lower expression of A20 in glucose treated microglia, m^6^A RIP assay was performed to detect the m^6^A modification level of *A20* in BV2 cells. Results showed that the m^6^A modification level of *A20* in the glucose treated microglia was observably higher than that in the control microglia (**Figure 6A**). m^6^A modification was determined by m^6^A-related methyltransferase METTL3 and METTL14, as well as m^6^A-related demethylases FTO and ALKBH5. Western blotting results showed that glucose treatment resulted in the decrement of ALKBH5 expression level in microglia, and the METTL3, METTL14 and FTO kept unchanged (**Figure 6B**). Lentiviruses encoding a short hairpin RNA for ALKBH5 and ALKBH5 overexpression lentivirus were also constructed. Inhibiting ALKBH5 in microglia decreased their A20 expression, and on the contrary increasing ALKBH5 up-regulated the A20 expression in microglia (**Figures 6C, E**). Besides, the m^6^A modification level of A20 mRNA was reduced by OE-ALKBH5 but increased by Lv-ALKBH5 (**Figures 6D, F**). Moreover, the degradation rate of *A20* mRNA in the glucose treated microglia was faster than that of the control microglia (**Figure 6G**). Inhibiting ALKBH5 accelerated the degradation rate of *A20* mRNA, and increasing ALKBH5 expression protected the *A20* mRNA from degradation (**Figure 6H**). Moreover, the luciferase activity of the reporter plasmid with mutant 2, rather than mutant 1 and 3, in *A20* 3’UTR was restored to the equal level of the control group. (**Supplementary Figure 2**).

## Discussion

Diabetic retinopathy is one of the most common microvascular complications of diabetes, which is characterized by retinal angiogenesis, leakage and ischemia (23). Considered as microvascular disease, DR was demonstrated to have a tight relationship with retinal inflammation in recent studies (24). What`s more, increased leukocyte entrapment was observed in the retinal vasculature three days after STZ induction in rats, indicating that inflammation came into being at the very early stage of diabetic retinopathy (25). Clarifying the mechanism of retinal inflammation may provide novel insight into the early diagnosis and intervention for DR.

In this study, we demonstrated that retinal microglia tended to polarize into M1 inflammatory type rather than M2 anti-inflammatory type in the diabetic model. These results were determined by not only the qualitative index of immunofluorescence assay in tissue levels, but also the quantitative index of flow cytometry assay and qRT-PCR assay in cells levels. Besides, the percentage of M1 microglia increased gradually, and that of M2 microglia decreased with glucose treatment from 0 to 100 mM. To investigate the mechanism in deep, RNA transcriptome sequencing data revealed differentially gene expression profiles of glucose treated microglia. Therein, several inflammatory signal pathways were enriched, and a critical molecular A20 was identified to negatively regulate the M1 polarization of microglia. Intraocular injection of A20-overexpression lentiviruses significantly rectified the enhanced M1 retinal microglia polarization of the diabetic model. In terms of mechanism, lower expression of ALKBH5 led to higher m^6^A modification level and faster degradation rate of *A20* mRNA, which at last resulted in decreasing A20 expression in retinal microglia of diabetic retinopathy.

Retinal microglia, a kind of tissue-resident macrophage, was the major inflammatory cell in the development of diabetic retinopathy (26). With different stimulation in a physiological or pathological condition, microglia polarize into M1 pro-inflammatory type or M2 anti-inflammatory type, which exhibit various functions in the retina (27). Unbalanced polarization or function of microglia not only leads to retinal degenerative but also inflammatory (28, 29). Recently, a study showed that M1 phenotype of microglia was found in neovascularization (NV) tufts in both central and peripheral retina, which contributed to the inflammatory environment of retinopathy (30). Moreover, M1 type microglia also affects the retinal neurons causing neurodegeneration which increasing apoptosis and thinning of nerve fiber layer, leading to visual defects (31).

In this study, we determined that unbalanced polarization of retinal microglia, manifesting as higher M1 percentage but lower M2 percentage, arose with the induction of diabetic rat model *in vivo* and with the glucose treatment *in vitro*. Similarly, previous researches showed that a high concentration of glucose induced the inflammatory phenotype of microglia with pro-inflammatory cytokines and chemokines causing chronic inflammation (9, 32, 33). Therefore, we suggest that high glucose conditions cause enhanced M1 polarization of microglia, which caused retinal inflammation and participated in the development of diabetic retinopathy.

Microglia polarization and function are under the regulation of various kinds of factors including glucose or inflammatory cytokines (33, 34). To illuminate the mechanism of enhanced M1 retinal microglia polarization in diabetic retinopathy, we perform transcriptome sequencing of high glucose treated microglia. Differentially expressed mRNA profile was identified and enriched in many inflammation related categories and signal pathways, indicating a pro-inflammatory status of microglia under high glucose conditions. Among these differentially expressed mRNA, lower expression of A20 is determined in glucose treated microglia. Previous studies have shown that A20 plays a critical role in the functional regulation of microglia (15, 35). A recent study further demonstrated that A20 promoted the M2 polarization of microglia (36). In our research, we found that in the *in vitro* experiment, inhibiting A20 expression promoted the M1 polarization of microglia and increasing A20 expression showed inverse results. Moreover, in the *in vivo* experiment, injecting the A20-overexpression lentiviruses also decreased the M1/M2 polarization ratio in the retina of diabetic rats. These results suggest that A20 negatively regulated the M1 proinflammatory of microglia. A20 is well known as an anti-inflammatory molecular, abnormal expression of A20 leads to the development of various kinds of diseases (37). Therefore, we concluded that a lower level of A20 expression could be one of the critical reasons for the inflammation microenvironment in diabetic retinopathy.

The mechanism of decreasing expression of A20 in diabetes retinal microglia is the key issue remaining to be addressed. Now that A20 expression firstly changed at the mRNA level, it is reasonable to assume that RNA modification may contribute to this process. N^6^-methyladenosine, the most common mRNA modification pattern in cells, is widely involved in the gene expression and function regulation of microglia (38, 39). Recently, several studies reported that m^6^A modification may contribute to the pathogenesis of diabetic retinopathy (19). Herein, we found that the m^6^A modification level of A20 was much higher in the glucose treated microglia, leading to the lower stability but faster degradation rate of A20 mRNA. M^6^A modification is under the control of methylase including METTL3 and METTL14, and demethylase including FTO and ALKBH5 (16). Zha`s study showed that METTL3 protected the high glucose treated retinal pigment epithelium cells from pyroptosis (40). In our study, we found that the expression of ALKBH5, rather than other m^6^A related methylase and demethylase, decreased after glucose treatment. Besides, regulating the ALKBH5 level determined that it reduced the m^6^A modification level, enhanced the mRNA stability and then increased the expression of A20. These results demonstrated that high glucose conditions resulted in enhanced M1 microglia polarization through ALKBH5 mediated m^6^A modification of A20.

Developing new treatments is the research hotspot of diabetic retinopathy nowadays. Even though the anti-VEGF therapy revolutionized the treatment of diabetic retinopathy but a significant proportion of patients, particularly those with sight-threatening macular edema, failed to respond. Furthermore, heavy economic burden, frequent injections related to endophthalmitis and other side effects indicate the requirement for investigating novel therapy (41, 42). With the discovery of the importance of inflammation in the pathogenesis of diabetic retinopathy, treatment targeting inflammation also shows outstanding effects. It has been shown that targeting A20 could exhibit therapeutics effects on inflammation and related diseases (43). Consistently, we showed that intraocular injection of A20-overexpression lentiviruses significantly reduced the M1 proinflammatory polarization of microglia, which could alleviate the inflammation level of the retina. This result suggests that A20 could be a promising therapeutic target for diabetic retinopathy, which still needs more support from basic and clinical researches.

In conclusion, we demonstrate that ALKBH5 mediated m^6^A modification of A20 regulated microglia polarization, which provides new insight into both the pathogenesis and the clinical intervention for diabetic retinopathy. Some limitations still exist, such as the expression level of A20 in patients with diabetic retinopathy and its clinical effect for these patients, which should be addressed in further researches.

## Data Availability Statement

The original contributions presented in the study are publicly available. This data can be found here: https://www.ncbi.nlm.nih.gov/bioproject/PRJNA770987/).

## Ethics Statement

The animal study was reviewed and approved by Institutional Animal Care and Use Committee of Sun Yat-Sen University, Guangzhou, China.

## Author Contributions

PW and LH contributed to designing research studies. TC and WZ contributed to conducting experiments and writing the manuscript. CW, XD, FY, YS, and JH contributed to analyzing data. All authors contributed to the article and approved the submitted version.

## Funding

This study was financially supported by the Guangdong Basic and Applied Basic Research Foundation (2019A1515110012), Natural Science Foundation of Guangdong Province (2021A1515010372), and Medical Scientific Research foundation of Guangdong Province, China (A2019117).

## Conflict of Interest

The authors declare that the research was conducted in the absence of any commercial or financial relationships that could be construed as a potential conflict of interest.

The reviewer WS declared a shared affiliation with the authors to the handling editor at the time of review.

## Publisher’s Note

All claims expressed in this article are solely those of the authors and do not necessarily represent those of their affiliated organizations, or those of the publisher, the editors and the reviewers. Any product that may be evaluated in this article, or claim that may be made by its manufacturer, is not guaranteed or endorsed by the publisher.

## Supplementary Material

Supplementary Figure 1 Function efficiency of Lv-A20 and OE-A20. **(A)** Lv-A20 inhibited the A20 mRNA level compared to the Lv-NC. **(B)** Lv-A20 inhibited the A20 protein level compared to the Lv-NC. **(C)** OE-A20 increased the A20 mRNA level compared to the OE-NC. **(D)** OE-A20 increased the A20 protein level compared to the OE-NC. Values are presented as the mean ± SD. *** indicates P<0.001.

Supplementary Figure 2 The binding target site of *A20* mRNA for ALKBH5. The luciferase activity of the mutant 2 group was restored to that of the control group. The luciferase activity of the mutant 1 and 3 groups, the same to the wild-type group, were significantly lower compare to that of the control group. Values are presented as the mean ± SD. *** indicates P<0.001.
