# Supplementary figures and images for: ALKBH5-Mediated m6A Modification of A20 Regulates Microglia Polarization in Diabetic Retinopathy
Source: Front Immunol. 2022 Mar 1;13:813979. doi: 10.3389/fimmu.2022.813979 (PMC8920977; doi:10.3389/fimmu.2022.813979)

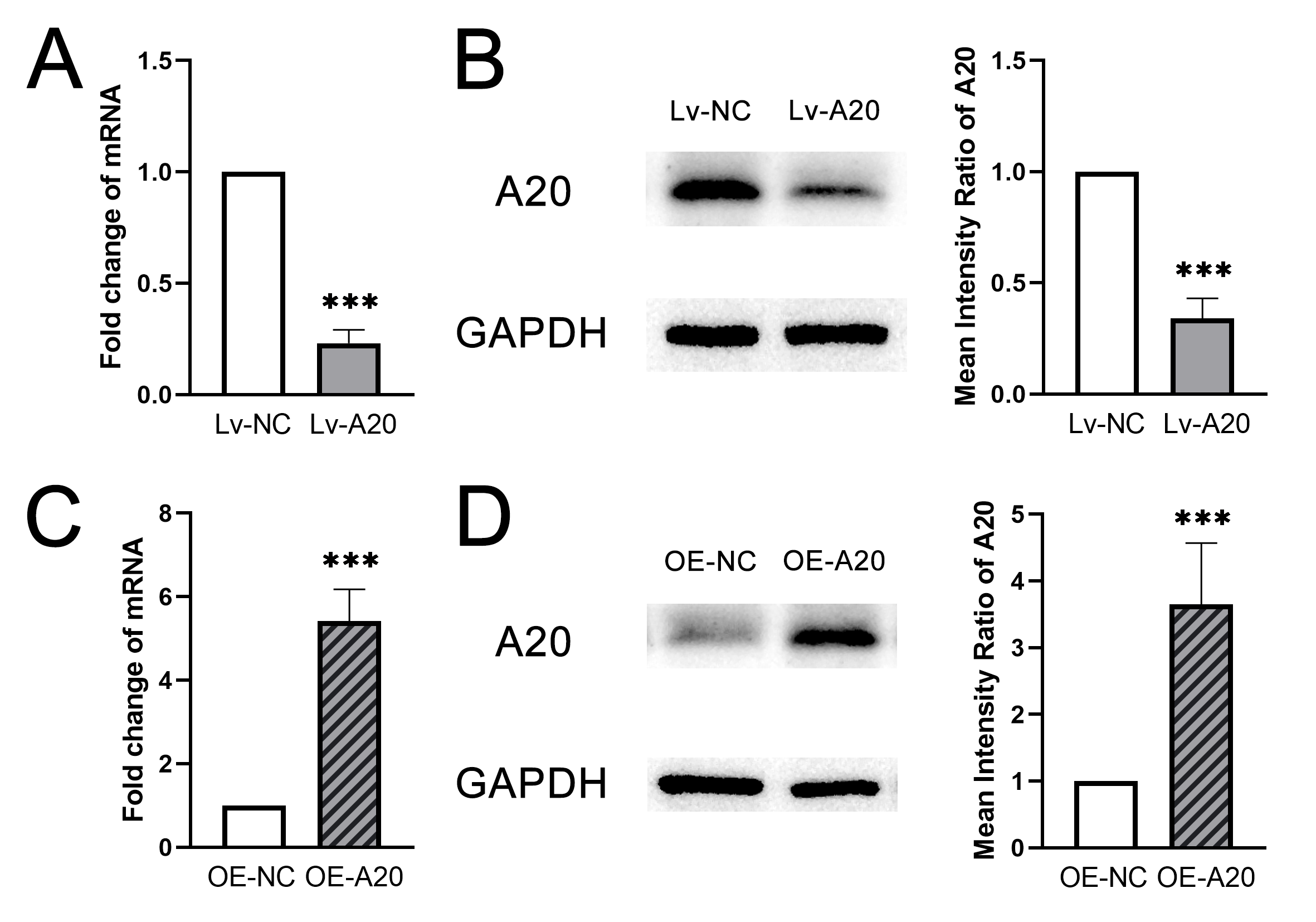

Supplement: Supplementary Figure 1 — Function efficiency of Lv-A20 and OE-A20. (A) Lv-A20 inhibited the A20 mRNA level compared to the Lv-NC. (B) Lv-A20 inhibited the A20 protein level compared to the Lv-NC. (C) OE-A20 increased the A20 mRNA level compared to the OE-NC. (D) OE-A20 increased the A20 protein level compared to the OE-NC. Values are presented as the mean ± SD. *** indicates P<0.001. [file Image_1.tif]

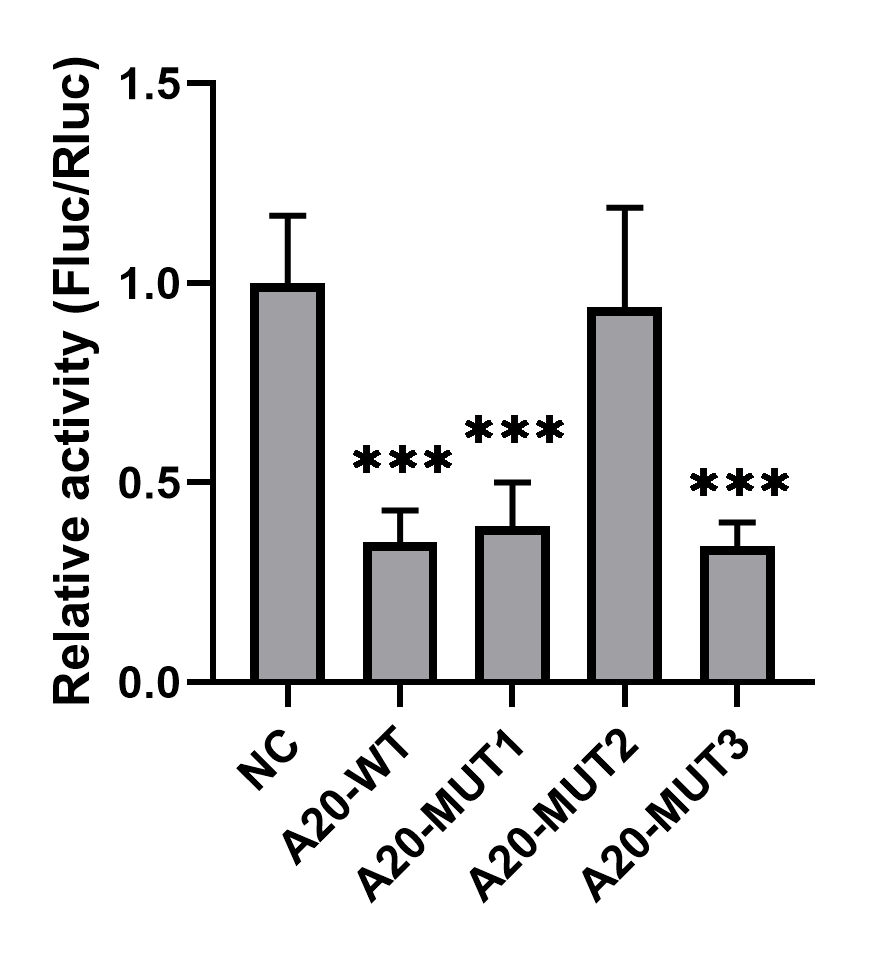

Supplement: Supplementary Figure 2 — The binding target site of A20 mRNA for ALKBH5. The luciferase activity of the mutant 2 group was restored to that of the control group. The luciferase activity of the mutant 1 and 3 groups, the same to the wild-type group, were significantly lower compare to that of the control group. Values are presented as the mean ± SD. *** indicates P<0.001. [file Image_2.tif]
