# Supplementary material for: ALKBH5-Mediated m6A Modification of A20 Regulates Microglia Polarization in Diabetic Retinopathy
Source: Front Immunol. 2022 Mar 1;13:813979. doi: 10.3389/fimmu.2022.813979 (PMC8920977; doi:10.3389/fimmu.2022.813979)
Supplement: Supplementary file 3 [file Table_1.docx]

**Supplemental Table 1 Primers for qRT-PCR**

| **Gene** | **Species** | **Forward primer**  **(5`-3`)** | **Reverse primer**  **(5`-3`)** |
| --- | --- | --- | --- |
| *GAPDH* | Rat | CCGCATCTTCTTGTGCAGTG | CGATACGGCCAAATCCGTTC |
| *IL-1β* | Rat | AGGCTTCCTTGTGCAAGTGT | CTTTTGGGGTCTGTCAGCCT |
| *IL-6* | Rat | GAGCCCACCAGGAACGAAA | AACTGGCTGGAAGTCTCTTGC |
| *TNF-α* | Rat | GATCGGTCCCAACAAGGAGG | CTTGGTGGTTTGCTACGACG |
| *IL-4* | Rat | TCCACGGATGTAACGACAGC | CGTTCTCCGTGGTGTTCCTT |
| *IL-10* | Rat | TCCGGGGTGACAATAACTGC | TTGGCAACCCAAGTAACCCTTA |
| *TGF-β* | Rat | CTGCTGACCCCCACTGATAC | AGCCCTGTATTCCGTCTCCT |
| *GAPDH* | Mouse | AGGTCGGTGTGAACGGATTTG | GGGGTCGTTGATGGCAACA |
| *IL-1β* | Mouse | GAAATGCCACCTTTTGACAGTG | TGGATGCTCTCATCAGGACAG |
| *IL-6* | Mouse | CTGCAAGAGACTTCCATCCAG | AGTGGTATAGACAGGTCTGTTGG |
| *TNF-α* | Mouse | CAGGCGGTGCCTATGTCTC | CGATCACCCCGAAGTTCAGTAG |
| *IL-4* | Mouse | CCCCAGCTAGTTGTCATCCTG | CAAGTGATTTTTGTCGCATCCG |
| *IL-10* | Mouse | GCTCTTACTGACTGGCATGAG | CGCAGCTCTAGGAGCATGTG |
| *TGF-β* | Mouse | CCACCTGCAAGACCATCGAC | CTGGCGAGCCTTAGTTTGGAC |
